# In vivo and in vitro efficacy of a single dose of albendazole against hookworm infection in northwest Ethiopia: open-label trial

**DOI:** 10.1186/s41182-021-00308-0

**Published:** 2021-03-20

**Authors:** Wolelaw Bezie, Mulugeta Aemero, Yalewayiker Tegegne, Tegegne Eshetu, Ayenew Addisu, Meseret Birhanie, Adane Derso, Ayalew Jejaw Zeleke

**Affiliations:** 1 Adet primary Hospital Laboratory, Adet, Ethiopia; 2 Department of Medical Parasitology, School of Biomedical and Laboratory Sciences, College of Medicine and Health Sciences, University of Gondar, P.O.Box: 196, Gondar, Ethiopia

**Keywords:** Hookworm, Single dose, Albendazole, In vivo, In vitro, Efficacy

## Abstract

**Background:**

Control of hookworm and other soil-transmitted helminth infections primarily relies on preventive chemotherapy using a single dose of albendazole/mebendazole drugs on high-risk groups. Herein, the efficacy of a single dose (400 mg) of albendazole (ALB) was investigated both in vivo and in vitro model in northwest Ethiopia.

**Methods:**

An open-label, single-arm clinical trial was conducted to assess anti-hookworm effect of albendazole. Stool samples were collected and examined using McMaster and Harada-Mori filter paper culture. Eligible hookworm-infected patients were treated with a single dose of ALB. After 14–21 days post-treatment, stool samples were also taken again and re-examined using the abovementioned technique. Egg reduction rate (ERR) and larval motility were used as a therapeutic outcome measure. An independent *t* test was used to compare the mean difference in egg counts, and probit analysis was performed for calculating the lethal concentration dose of albendazole*. P* value < 0.05 at 95% CI was considered statistically significant.

**Results:**

A total of 70 participants had completed the drug efficacy study. The efficacy of ALB against hookworm in terms of CR and ERR was 87% and 93%, respectively. Participants who had not eaten one or more hours prior to treatment had higher CR than those who had eaten within 1 h before treatment (97.4% vs 74.2%), while individuals with heavy infection intensity had a lower post-treatment ova clearing rate than those who were with light infection intensity (43% vs 94.6%). The in vitro larvicidal effect of ALB was 63–93% after applying 50–250 μg/ml concentration of ALB solution. The LC50 and LC99 were 152 μg/ml and 573 μg/ml, respectively.

**Conclusion:**

A single dose of albendazole was found to be effective for treating hookworm infections according to WHO anthelminthic evaluation standard in the study area. Preventive chemotherapy might therefore be extended to risk groups, with proper continuous monitoring of its efficacy to strengthen and keep the ongoing control and prevention measures one step ahead.

**Trial registration:**

This trial is retrospectively registered with www.pactr.org, number PACTR202010511829332 on October 26, 2020.

## Background

Soil-transmitted helminths (STH) infections (*Ascaris lumbricoides*, hookworm, and *Trichuris trichiura*) are the most common infections worldwide and disproportionately affect the poorest and most deprived communities with some of the highest burdens occurring in sub-Saharan Africa [1, 2]. Hookworms (*Ancylostoma duodenale* and *Necator americanus*) are the most important among the three big STHs and are responsible for 845,000 disability-adjusted life years (DALYs) among the estimated 500 million infected people worldwide [3, 4]. Besides, it accounts for $7.5 billion to $138.9 billion productivity lost annually [3]. The burden of the disease is mainly attributed to morbidity, particularly anemia and malnutrition, rather than the mortality it causes [5, 6]. Hookworm infection is a recognized major cause of gastrointestinal blood loss and leads to iron, energy, protein, and zinc deficiency, particularly pronounced in children and pregnant women [7, 8]. As a result, it causes more subtle chronic health problems such as growth retardation, as well as intellectual and cognitive impairment in children, and adverse maternal-fetal outcomes in pregnant women [9, 10].

Although hookworm causes such adverse health outcomes, the magnitude of the problem has long been neglected because these outcomes rarely occur at a young age, due to its insidious and chronic nature [11]. Nowadays, unprecedentedly, many governmental agencies, donors, and international organizations have initiated to control the impact of hookworm and other neglected tropical diseases through improved water and environmental sanitation to reduce transmission and administer preventive chemotherapy to the three high-risk groups: preschool-aged children (PSAC), school-aged children (SAC), and women of reproductive age (WRA) to minimize adverse effects following high infection intensity [2, 11–14]. Despite the control approaches initiated by incorporating improved water and environmental sanitation strategies, the current focus has been exclusively on the approach of preventive chemotherapy with benzimidazole agents (albendazole/mebendazole) targeting SAC mainly in developing countries through the education sector [12].

Hence, it would not reach for those who do not regularly attend the school, nor will it address the potentially significant adult reservoirs of infection within the targeted communities. In addition, the widespread distribution of benzimidazole agents may lead to resistance in human STH, the impact of which on global control efforts has not been carefully assessed. Since this drug has been used for more than three decades and a comparison of efficacy measures over time indicates a decreasing trend, although resistance has not yet been documented in human use. Experience from the veterinary sector demonstrated that anthelminthic drug resistance developed after years of large-scale monotherapy. If we are going to wait until resistance is clinically detected in humans, it will be too late to respond. In view of insufficient efficacy, coupled with the potential for resistance emergence from long-term use, there is an urgent need not only to develop new therapies against STH infections but also to optimize current treatment regimens [15–17].

Moreover, despite studies that revealed an inconsistent efficacy level of albendazole (400 mg) in terms of fecal egg reduction rate (FECRT/ERR) against hookworm, most of these studies assured its reduced efficacy status [17–20]. Although ERR is currently the only applicable and recommended unit to determine anthelminthic outcomes in humans, it lacks sensitivity when less than 25% of the helminth population carries a resistance gene, as is the case in the veterinary setting [21]. Therefore, the use of multiple diagnostic approaches to assess the outcome of anthelminthic is essential.

In addition, it is essential for the success of available control programs to track the efficacy of these drugs and to monitor parasite populations for possible rise of anthelminthic resistance (AR). Therefore, this study was designed to determine the current status of the efficacy of albendazole (400mg) against hookworm using both in vivo and in vitro methods.

## Methods

### Study design

An open-label, single-arm clinical trial was conducted from February 1, 2020, to March 30, 2020, at Adet Primary Hospital in Yilmana Denssa district, Northern Ethiopia. All outpatients who visited Adet Primary Hospital during the study period were invited to be enrolled in this study. This trial is retrospectively registered with www.pactr.org, number PACTR202010511829332 on October 26, 2020.

### Intervention and origin of the drug

This study was primarily designed in a single treatment arm to assess the efficacy of a single dose of albendazole (400 mg) in vivo, while six different tube culture methods: one tube culture for positive control and the remaining five-tube culture with a different concentration of albendazole solutions were used to assess the in vitro larvicidal effect of the drug. The solid tablet of albendazole brand (Bendex, India, Cipla Limited, batch no: Cqt5djo11) with a label claim of 400 mg/tablet was obtained from Adet Primary Hospital in Yilmana Denssa district northwest Ethiopia, while the syrup albendazole drug (Bendex, India, Cipla Limited, Batch no: A390342) with a label claim of 10ml suspension was purchased from a private community pharmacy in Injibara town, Ethiopia.

### Eligibility criteria and sample size

All hookworm-infected outpatients who agreed to comply with the study procedures, including provision of two adequate stool samples at baseline and at post-treatment follow-up assessments (approximately 2 weeks later). Patients whose age was above 2 years and who had a signed informed consent form from the participant him/herself or legal representative were included, as well as patients who had no underlying health problems. On the other hand, individuals who were under anthelminthic treatment within the last 6 weeks prior to data collection, pregnant women, participants taking medication with a known interaction (e.g., for albendazole: cimetidine, praziquantel, and dexamethasone), diarrhea, or vomiting at the time of first sampling and during treatment were also applied as exclusion criteria.

The intended sample size was calculated according to the WHO drug efficacy guideline [22]. A minimum of 50 participants positive for each of the targeted parasites was sufficient to evaluate the efficacy of the investigated drug. To obtain the minimum number of hookworm-positive cases, we used the following assumptions: a conservatively estimated compliance rate of 80% and an estimated prevalence of hookworms in the study area of 22% [23]. Additionally, considering the potential loss to follow-up, a non-response rate of 20% was added. Finally, 340 patients were considered for screening to get the minimum required sample size. A convenience sampling technique was used until the required sample size was reached.

### Data collection and laboratory procedures

Before the commencement of the study, training was given to laboratory professionals on how to collect data from study participants and how to assure the quality of data while they were collecting the data. Afterward, a questionnaire that was developed according to the WHO efficacy assessment guideline to collect participants’ socio-demographic characteristics and expected factors that might affect the efficacy of the drug were administered to the study participant. A sterile and leakproof stool container labeled with the participants’ unique identification number was given to each study participant and asked to provide approximately 10 mg of a fresh stool sample. Participants were reminded to avoid any possible contamination while they were collecting the required stool sample. Each stool sample was immediately examined in the hospital laboratory to avoid any possible sample delay.

Direct wet mount and McMaster diagnostic techniques were used to detect the presence of hookworm eggs or larvae in the stool. The McMaster diagnostic technique was used to quantify fecal egg intensity, which is a standard reference method for evaluating drug efficacy in veterinary parasitology and has recently been used in human parasitology [22, 24]. In addition, microscopically confirmed stool samples for hookworm were placed on Harada-Mori filter paper in Adet Hospital Laboratory. Then, the stool culture was transported to Bahir Dar Amhara Public Health Institute (APHI) to assess the growth of hookworm larvae. Moreover, the in vitro larvicidal effect of albendazole was assessed by applying different Albendazole concentrations.

### In vitro development of hookworm larva

Fresh stool samples were collected and cultured for 7–10 days using the Harada-Mori test tube filter paper culture technique. One gram of feces was smeared in narrow 13 × 120 mm filter paper strip and placed in a 15-ml conical centrifuge tube containing about 5-ml distilled water. The distilled water was placed under the fecal spot (smeared feces). The feces were not soaked or washed into the bottom of the test tube. The test tubes were labeled using the marker tape and stood vertically in the test tube rack. The culture tube was covered with a tube lid and kept in at incubator at 25–28 °C for a maximum of 7–10 days and examined under low power magnification (l0×) for emerging of hookworm larvae [25, 26].

### Drug administration and follow-up

After performing all required parasitological and individual participant data collection, participants who had microscopically confirmed hookworm infections underwent further clinical examination to ensure their eligibility for this study by senior nurses. Participants who were infected with other parasitic infections were linked to a physician to have them treated with appropriate medications according to the treatment guideline. On the other hand, eligible participants who had microscopically confirmed hookworm infections were treated with a single dose of albendazole (400mg). Participants were informed of the side effects to be expected after albendazole administration such as headache, fever, nausea, vomiting, stomach pain, dizziness, and transient hair loss.

Each participant was also reminded to report post-treatment medical complaints to the principal investigator by calling or visiting the nearby health facility. Each treated participant was asked to return at after 14–21 days post-treatment to provide a second stool sample. During the follow-up of the stool samples’ collection period, participants were asked about any medical discomfort following administering the drug. A participant who vomited within 4 h of drug administration or a participant who had diarrhea at the time of first sampling was not included in the final analysis. The McMaster diagnostic technique was used to detect and quantify the number of fecal eggs during the follow-up period. Participants who remained infected with hookworm and other STH have received a triple dose of albendazole (100mg for three consecutive days).

### In vitro assessment of the larvicidal effect of albendazole

Five different concentrations of albendazole solution were used in this study (50 μg/ml, 100 μg/ml, 150 μg/ml, 200 μg/ml, and 250 μg/ml). Six tube cultures were prepared for each microscopically confirmed hookworm stool sample. Tube 1 served as positive control and the other five were used to assess the effect of the drug on hookworm larvae with different concentrations of albendazole. Once the larva was confirmed as hookworm parasite, four milliliters of various concentrations of albendazole or distilled water (as a control) was added to the bottom of each tube at 25 °C. We used 20 mg/ml of 20 ml albendazole syrup to prepare these albendazole solutions [27]. Then, 1 ml of 20mg/ml syrup was diluted by 49-ml distilled water and it gave 50 ml of 400 μg/ml ABZ. The test concentrations (250 μg/ml, 200 μg/ml, 150 μg/ml, 100 μg/ml, and 50 μg/ml) were prepared as follows; first of all, 3.5ml, 3ml, 2.5ml, 2ml, and 1.5ml from 5ml of the supernatant of the culture containing hookworm larvae were discarded. The remaining volume of the culture (the bottom of the tube) was 1.5 ml, 2ml, 2.5ml, 3ml, and 3.5ml, respectively. Then, 2.5 ml, 2ml, 1.5ml, 1ml, and 0.5 ml were taken from 50ml of 400 μg/ml and had been added on the respected volumes of the cultures, respectively. This finally gave us 4ml of 250 μg/ml, 200 μg/ml, 150 μg/ml, 100 μg/ml, 50 μg/ml, respectively. Thereafter, all of the cultures were incubated for 48 h at 25–28 °C and the effect of these albendazole solutions on the motility of hookworm larvae were observed microscopically.

### Outcome measures

Fecal egg reduction rate (ERR), which is the primary outcome measure, was used to assess the therapeutic outcome of albendazole against hookworm infection. Besides, cure rate (CR) of albendazole was used qualitatively as a secondary outcome measure against hookworm infection.

The CR and ERR were calculated using the following mathematical formula;

$$ \mathrm{CR}=\frac{\mathrm{Number}\ \mathrm{of}\ \mathrm{participants}\ \mathrm{infected}\ \mathrm{with}\ \mathrm{Hookworm}\ \mathrm{who}\ \mathrm{were}\ \mathrm{cured}}{\mathrm{number}\ \mathrm{of}\ \mathrm{infected}\ \mathrm{subjects}\ \mathrm{who}\ \mathrm{were}\ \mathrm{treated}}\times 100\% $$

$$ \mathrm{ERR}=\left[1-\frac{\mathrm{mean}\ \mathrm{at}\ \mathrm{follow}\hbox{-} \mathrm{up}}{\mathrm{Mean}\ \mathrm{at}\ \mathrm{baseline}}\right]\times 100\% $$

The drug efficacy status in terms of CR and ERR was evaluated according to the WHO guideline [22]. Moreover, the motility of the larva was used to assess the larvicidal effect of the drug using different concentrations.

### Quality control

The reliability of the study finding was ensured by applying quality control measures on the whole process of the laboratory work (pre-analytical, analytical, and post-analytical quality control steps were followed). Known negative and positive control samples were used to check the functionality of the microscope and selected culture media used during the study period. Moreover, data collectors and laboratory technicians were supervised by the principal investigators while they were performing their assigned tasks.

### Data management and analysis

All registered data in the data collection sheet during the study period were transferred to Epi data software to check its completeness and clearance and transferred to SPSS software for further statistical analysis. Only participants who had completed data set in both baseline and follow-up survey (per-protocol analysis) were included in the final efficacy assessment. Descriptive statistics were used to analyze the socio-demographic characteristics of the study participants. Pearson’s chi-square was also used to show the association of study variables.

Moreover, an independent *t* test was performed to compare the mean difference in fecal egg count and probit analysis was used for calculating the LC dose of albendazole. *P* value < 0.05 at 95% CI was considered statistically significant.

## Results

### Socio-demographic characteristic and prevalence of hookworm

A total of 340 individuals were involved during baseline screening. Of these, 194 (57%) were males. The overall prevalence of hookworm infection among the outpatients in the study area during the study period was 25% (85/340). Fortunately, all 85 hookworm-infected participants fulfilled all the required eligibility criteria of the study and were treated with a single dose of albendazole (400 mg). Among participants who received the treatment, only 70 participants had completed the follow-up period and included in the final efficacy analysis. The remaining 15 treated participants were absent during the follow-up data collection time frame.

The mean age of participants who enrolled in the final analysis was 38.74 ± 16.2 SD years with a minimum of 5 years and maximum of 73 years. Socio-demographic characteristics of participants are summarized in Table 1.

**Table 1:** Socio-demographic characteristics of study participants’ attending Adet Primary Hospital from February 1 to March 30, 2020 EC

	Variables	Frequency	Percent (%)
Sex	Male	38	54.3
	Female	32	45.7
Age	≤ 15	5	7
	16–30	20	28.6
	31–45	22	31.4
	>45	23	33
Residence	Urban	24	34.4
	Rural	46	65.6
Educational status	Diploma and above	7	10
	Secondary level	12	17.1
	Primary level	17	24.3
	No formal education	34	48.6
Marital status	Single	14	20
	Married	51	73
	Other	5	7
Occupation	Farmer	42	60
	Merchant	4	5.7
	House wife	10	14.3
	Student	14	20

### In vivo efficacy of a single dose of albendazole against hookworm infection

After 14 days of post-treatment, CR was found to be 87% (95% CI 78.6–94.3%), while the ERR was 93% (95% CI 90–97%). In terms of infection intensity, out of 70 study participants were involved in the final analysis, 37 (53%), 26 (37%), and 7 (10%) of them had light, moderate, and heavy infection intensity, respectively.

Despite 87% (61/70) of participants were cured following a single dose of albendazole, 11.5% (8/70) and 1.4% (1/70) of participants remained under light and moderate intensity of infection, respectively. The arithmetic mean of fecal egg count (FEC) of hookworm infection at baseline was 2027epg (95% CI 1764–2285) with a minimum of 500 EPG and a maximum of 9700 EPG.

The intensity of hookworm infections in terms of arithmetic means was reduced to 142 EPG (95% CI 48–265) with a minimum of 0 EPG and a maximum of 2000 EPG following 2 weeks post-treatment. A statistical significant difference in mean egg reduction (1885 EPG) was observed following treatment (95% CI 1580–2190, (*P* ≤ 0.001)). Infection intensity at baseline was found to be the most significant factor with a considerable effect on the CR of hookworm. Most of the study participants who had heavy infection intensities were not cured following a single oral dose of albendazole.

### Associated factors that affect albendazole efficacy against hookworm

In our finding, participants’ age, gender, area of residence, types of food, water source, and co-infection were not associated with the efficacy of the drug. However, baseline fecal worm intensities and feeding time were significantly associated with the efficacy status of the drug. Of the study participants who were with heavy infection intensities at the baseline investigation, 43% were cured after treatment, while 94.6% of study participants who were under light infection were cured (*X*^*2*^ =14.12; *P*<0.001). Moreover, the CR was higher (97.4%) for those of the study participants who had taken the drug 1 h before meal. In contrast, study participants who had taken the drug 1 h after meal had a lower CR of 74.2% (*X*^*2*^ =8.32; *P*=0.008).

### In vitro larvicidal effect of albendazole on hookworm

**Fig. 1:**
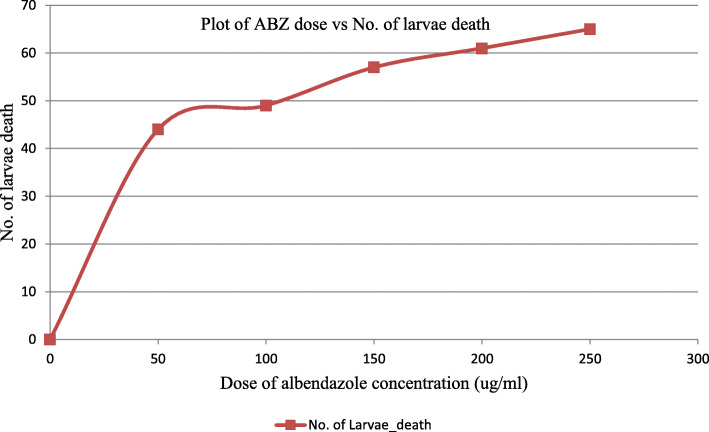
In vitro dose response curve of different albendazole concentration against hookworm larvae death

After the application of different concentrations of albendazole on the larvae stage, the lowest and highest mortality rates were observed at 50 and 250 μg/ml of the drug, respectively. The 50 μg/ml of albendazole results in a 57% mortality rate, while the 250 mg/l of the drug resulted in 93% (65 of 70) of the larval death. The LC99 values against the labor parasite larva were 573 μg/ml (Table 5). This study showed that the larval mortality rate increased with an increasing albendazole concentration (Fig. 1).

## Discussion

Although hookworm is being treatable and preventable, its effect continues to have a devastating impact on peoples’ health in Ethiopia. The national control program is successfully mapped to achieve the elimination of those parasitic diseases in 2020 and aim to attain transmission break by 2025 using either a single dose of albendazole (400mg) or mebendazole (500mg) [28, 29]. As a result, a single dose of albendazole (400mg) is one of the extensively and frequently used anthelminthic drug in the mass drug administration (MDA) campaign for treating hookworm and other STH infections throughout the country. Such frequent and extensive use of the drug may lead to the development of drug tolerance and resistance. Thus, it is a call for continuous monitoring of the therapeutic efficacy of a single dose of albendazole.

Although existing literature have revealed the inconsistencies and reduced therapeutic efficacy status of a single dose of albendazole against hookworm infections [17–19, 30, 31], our finding unlikely showed its better therapeutic outcome status in terms of ERR of 93% (95% CI 90–97%). This efficacy status of albendazole against hookworm in the current study is considered *satisfactory* therapeutic outcomes according to the WHO albendazole efficacy evaluation standard [22]. Such conflicting and inconsistent findings regarding the therapeutic outcomes of albendazole against hookworm infections might be related to the distinct brands of albendazole tested to assess its therapeutic outcomes. Available evidence assured that different brand formulation alters the bioequivalence of a certain drug due to the impact of used excipients and inactive substances that could modify the ability of the active drug component to go into solution [32]. Moreover, the efficacy of albendazole (400mg) in terms of ERR (93%) in this study was lower than the finding reported in Wondogenet, southern Ethiopia, ERR (99.8%) [33]. This discrepancy might be due to a difference in the laboratory method (procedure) used and variation in study participants. McMaster diagnostic technique was used in the current study, unlike in Wondogenet that was Kato-Katz method. Moreover, this study enrolled study participants whose age was greater than 2 years in addition to the school-aged children in contrast to the after mentioned study. Our finding also revealed lower efficacy status of albendazole against hookworm infections compared with a previous study conducted in Jimma, southwest Ethiopia [34]. The variation in the brand of albendazole might be the possible factor for the inconsistency of its therapeutic outcome. Hence, different brands of albendazole may contain distinct additive substances that facilitate treatment efficacy. In this study, each participant has received a single brand of albendazole, unlike the study done in Jimma. Similarly, the present finding showed a lower efficacy than a study conducted in three STH-endemic countries (Ethiopia, Lao PDR, and Tanzania) by using five laboratory diagnostic methods. The overall single dose of albendazole efficacy in Ethiopia and Lao PDR was within the range of 93.6 to 99.3% [35]. This inconsistency might be due to different diagnostic technique used in the two studies. Alternatively, our result (CR =87% (95% CI 78.6–94.3%) and ERR =93% (95% CI 90–97%)) was consistent with other study conducted from seven endemic countries including Ethiopia (87.8% and 94.8%) [36]. Moreover, this finding was consistent with studies conducted in Gabon (92%) and Tanzania (79.6 to 90.3%) [35, 37]. However, our study result was higher than studies conducted in Ghana, Leo PDR, and China to evaluate the efficacy of a single dose of albendazole on hookworm infections. The overall CR ranged from 35 to 69% and ERR 61 to 90.7% [17, 18, 38, 39].

Statistically significant difference in the reduction of mean egg count was observed between pre-and post-treatment periods [40]. Moreover, although there is a significant reduction of the mean FEC (1885 EPG) after treatment (95% CI 1580–2190, (*P* <0.000), the baseline infection intensity has significant effect on the overall efficacy status of the drug (Table 2) [36]. In this study, participants who were with heavy infection intensity at baseline had lower CR (43%) (*X*^*2*^ =14.12; *P*<0.001) than those who had light infection intensity with CR of (94.6%) after treatment. This could indicate that a single dose of albendazole may not be sufficient to clear hookworm during heavy infection intensity (Table 3).

**Table 2:** The baseline infection intensity and curative status of respondents

Infection intensity during baseline	Curative status of respondents				Total
	Cure		Non-cure		
	Count	Percent	Count	Percent	
Low	35	94.6%	2	5.4%	37/100%
Moderate	23	88.5%	3	11.5%	26/100%
Heavy	3	43%	4	57%	7/100%
Total	61	87%	9	13%	70/100%

**Table 3:** Hookworm infection intensity and egg reduction rates in pre- and post-treatment periods among study participants

Treatment status	Infection intensity, *n*(%)			Mean EPG	Egg reduction rate
	Light	Moderate	Heavy		
Pre-treatment	37(53)	26(37)	7(10)	2027	93%
Post-treatment	8(88.9)	1(11.1)	0(0)	142	

Although there is no recommended guideline regarding anthelminthic drug administration in relation to feeding time, the current study highlighted that it was found to be significant factor for the efficacy of the drug. This finding revealed that participants who took the drug 1 h after meal had low curative status (CR=72.4%) than participants who took the drug 1 h before meal (97.4%). Patients who had fasted an hour before treatment were significantly more likely to be cured of hookworm (*X*^*2*^ =8.32; *P*=0.008) (Table 4). This finding is supported by a study conducted among school children [17] and revealed children who have not eaten at least 6h before treatment had a cure rate of (CR= 90%), while, participants who did more recently had a cure rate of (CR =59%). This might be due to the simultaneous taking of food with the drug may limit the absorption and bioavailability of the active ingredients of the drug. In this regard, existing evidence in the veterinary area assured that not only feeding time but also food type and composition could significantly influence the pharmacokinetics and pharmacodynamics of albendazole drugs in association with its poor solubility nature of the drug. The reduced amount of food intake and longtime starvation significantly increase the bioavailability and pharmacokinetics of albendazole, respectively, and this might have led to significant increase in anthelminthic efficacy [41]. Moreover, the presence of food in the stomach affects the bioavailability of a certain drug by preventing the absorption process, and it is assured through the decrease C_max_ and increase T_max_ status [42].

**Table 4:** Associated factors with cure rate of hookworm among patients (*n*=70)

Variables		Outcome of treatment		Chi-square	*P* value
		Cured, *n*(%)	Not cured, *n*(%)		
Sex	Male	37(52.8)	2(2.90)	4.69	0.68
	Female	24(34.3)	7(10.0)		
Age in years	<15	3(4.3)	2(2.9)	3.7	0.29
	16–30	18(25.7)	2(2.9)		
	31–45	20(28.6)	2(2.9)		
	>45	20(28.6)	3(4.30)		
Feeding status	Fasting	55(78.6)	9(12.9)	0.968	0.62
	Non-fasting	6(8.6)	0(0)		
Co-infection	Yes	22(31.4)	4(5.70)	0.24	0.62
	No	39(55.7)	5(7.10)		
Infection intensity	Light	35(50)	2(2.9)	14.13	0.001
	Moderate	23(32.9)	3(4.3)		
	Heavy	3(4,3)	4(5.70)		
RX taking time	1 h before meal	38(54.3)	1(1.40)	8.33	0.008
	1 h after meal	23(32.9)	8(11.4		

Moreover, the present study has presented important findings about the in vitro larvicidal effect of albendazole using Harada Mori tube filter paper culture by applying different concentrations of albendazole solution on the larval stage of hookworm (Table 5). Following the application of the different concentrations of albendazole solution namely 50 μg/ml, 100 μg/ml, 150 μg/ml, 200 μg/ml, and 250 μg/ml, about 44 (63%; 95% CI 51.4–74.3%), 49 (70%; 95% CI 58.6–80%), 57 (81.4%; 95% CI 73–90%), 61 (87%; 95% CI 78.6–94.3%), and 65 (93%; 95% CI 87.1–98.6%) of the stool culture had observed with dead or non-motile larvae, respectively. In contrast, none of the larvae from the control groups neither died nor lost their viability. The 50% and 99% LC of albendazole against hookworm were 152 μg/ml and 573 μg/ml, respectively. This indicated a dose-response relationship as the concentration of albendazole increase; the larvicidal effect was also increased. In our finding, assessment of the in vitro larvicidal effect of albendazole suggest that the possibility of providing supportive evidence on the susceptibility of hookworm parasite following albendazole treatment. Moreover, the in vitro larvicidal assessment highlighted its importance for larva cultivation for several studies. Besides, it indicates its diagnostic role for hookworm infection.

**Table 5:** In vitro hookworm larva killing effect of albendazole

Dose response		# dead larvae	Estimated LC values and confidence limits			
Conc. (μg/ml)	# of exposed larvae		LC	Mean conc. (μg/ml)	95%CI	
					Lower	upper
50	70	44	5	59.9	0.16	111.9
			10	73.4	0.50	126
100	70	49	15	84.4	1.07	137
			20	94.3	1.94	146.5
150	70	57	50	152.3	24.37	201.5
			60	175.9	50.52	229.2
200	70	61	70	205.4	102.00	283.4
			75	223.7	139.20	346.1
250	70	65	80	246.1	176.70	480.1
			90	316.2	241.51	1557
Control	70	0	95	388.9	282.00	4557
			99	573.477	360	55823

Generally, despite a single dose of albendazole showed better efficacy outcome for treating light hookworm infection intensity, individuals with a heavy intensity of infection remained under question. The other interesting finding of this study is that the in vitro parasite cultivation may give us a green light to use it as an alternative diagnostic approach for estimating the outcomes of anthelminthic drugs. Although the in vitro model for assessing the efficacy status of albendazole could be considered as the strength of this study, optimization of the albendazole dose concentration was not standardized since there was neither a previous study nor a standard guideline. Hence, we recommend further investigation on the in vitro larvicidal effect of albendazole analysis using different types of albendazole solutions.

## Conclusion

In conclusion, the present study showed that single dose of albendazole is effective for the treatment of hookworm infections. As a result, our finding encourages the use of single dose of albendazole (400mg) as preventive chemotherapy with proper monitoring of its therapeutic outcome. In addition, the in vitro parasite cultivation may give us a green light to use it as an alternative diagnostic approach for estimating the outcomes of anthelminthic drugs. The study also added evidence regarding the significance of feeding time and baseline infection intensity on the therapeutic outcome of albendazole, and could be better if it take in to consideration during treatment program in the study area and other endemic setting.

## Data Availability

The data generated or analyzed during this study are included in this manuscript. Other data will be available from the corresponding author upon request.

## Abbreviations

ALB: Albendazole
AP: Agar plate
AR: Anthelminthic resistance
BZ: Benzimidazole
CR: Cure rate
DALYs: Disability-adjusted life years
EPG: Eggs per gram
ERR: Egg reduction rate
FEC: Fecal egg count
IPI: Intestinal parasitic infection
KK: Kato-Katz
LC: Lethal concentration
LMA: Larval motility assay
MDA: Mass drug administration
STH: Soil-transmitted helminths
WHO: World Health Organization
